# Identifying patterns of religiosity in adults from a large UK cohort using latent class analysis

**DOI:** 10.12688/wellcomeopenres.17969.2

**Published:** 2025-09-04

**Authors:** Isaac Halstead, Jon Heron, Carol Joinson

**Affiliations:** 1 The Centre for Academic Child Health, Population Health Sciences, Bristol Medical School, University of Bristol, Bristol, Gloucestershire, BS8 2BN, UK; 2 Population Health Sciences, Bristol Medical School, University of Bristol, Bristol, Gloucestershire, BS8 2BN, UK

**Keywords:** Religion, latent class analysis, ALSPAC, individual differences, religiosity

## Abstract

**Background:**

Religiosity is a complex, multifaceted construct, comprising a variety of beliefs and behaviours. Much of the previous research that seeks to quantify religiosity has made use of variable-centred approaches, which place individuals on a continuum of religiosity. However, alternative approaches provide a way to examine different *types* of religiosity, represented by heterogeneous classes of religious (non)belief. The aim of this study was to apply a person-centred approach to understanding religious beliefs.

**Methods:**

The present study used latent class analysis to identify classes of belief and non-belief in mothers (n=12429) and their partners (n=9953) from the Avon Longitudinal Study of Parents and Children, a large cohort study based in the UK. For this, we used a range of religious belief indicators. We also examined a number of socioeconomic factors, to identify differences between classes, using logistic regression.

**Results:**

We identified four different classes of religiosity which we named Highly Religious, Moderately Religious, Agnostic, and Atheist, with similar configurations in both men and women. We also found that class membership was associated with several socioeconomic factors.

**Conclusions:**

The findings provide an insight into different patterns of religiosity in adults in the UK. These classes could be used as exposures in future studies of religiosity and how it relates to a variety of outcomes in both mothers, partners, and their offspring.

## Introduction

Much thought has been applied to the conception of religiosity scales, with well over 100 different measures of religiosity and spirituality in existence ( Hill & Hood, 1999). Consequently, it can be difficult to typify religiosity. However, many of these measures tap into several consistent themes. Firstly, religiosity measurement generally appears to consist of three main components (e.g., Johnstone *et al.*, 2021; Koenig & Büssing, 2010; Tong & Yang, 2018; Worthington *et al.*, 2003); cognitive (beliefs about religion), affective (experiences of a religious nature, feeling the presence of the divine), and behavioural (attendance at church, prayer, meditation) ( Cornwall *et al.*, 1986). These components of religiosity are underpinned by religious motivations, typically considered in terms of intrinsic (‘religion as an end in itself’, expressed through prayer and other self-directed behaviours) and extrinsic religiosity (‘religion as a means to an end’, expressed through church attendance and other socially directed behaviours that often benefit the individual) ( Allport & Ross, 1967) (for examples of these constructs, see Bravo *et al.*, 2016; Hills *et al.*, 2005; Koenig & Büssing, 2010). The variety of measures available and different aspects of religion previously identified suggest that any single measure of religious beliefs is unable to accurately capture this complex construct.

Religiosity is often examined using variable-centred approaches that identify associations between variables, such as factor analysis or principal component analysis (e.g. Hills *et al.*, 2005; Pearce *et al.*, 2017), which assumes that quantitative differences exist between individuals in their levels of religiosity, but each item has the same meaning to different individuals. Measurements of religiosity that use this approach are, however, constrained to a high/low religiosity continuum (i.e., where people are only allowed to have varying degrees of religiosity, rather than differing *types* of religiosity). This fails to account for the many potentially contrary beliefs, feelings or behaviours that constitute religiosity. For example, it is conceivable that a deeply religious person may not attend a place of worship or believe that God personally intervenes to help them in times of trouble. These nuances are lost when examining religiosity from a variable-centred perspective. This suggests that a person-centred approach (that assumes items intended to examine a topic may have different meanings for different groups of people) to examining religiosity would provide an important insight into the way these different thoughts, feelings and behaviours associated with religiosity cohere in an individual. The present study provides an alternative approach to examining religiosity seen in similar papers, such as Major-Smith *et al.* (2022), which used a variable-centred approach.

When religiosity is examined with a person-centred approach, such as latent class analysis (LCA), interesting patterns of responding arise. For example, recent work found that a modest proportion of people that indicated they possess no religious belief (instead identifying as spiritual but not religious) were still likely to attend church and pray ( Tong & Yang, 2018). Additionally, work by Bravo *et al.* (2016) found a four-class solution that consisted of high and low religiosity classes, but also intrinsically motivated and questioning classes, which supports the heterogeneity of different types of religiosity. Finally, work by Vasilenko and Espinosa-Hernández (2019), found five latent classes that corresponded to high religiosity and different ways of practicing religion, such as privately (through prayer), publicly (through attendance), and purely affiliation (no indicators of religiosity barring a declaration of identifying as a particular faith). In combination, these findings indicate that religious beliefs and behaviours are a complex pattern of behaviours, and sometimes contradictory beliefs, which is often overlooked through traditional variable centred approaches.

Identifying different groups of religious belief is an important step in examining the ways that religiosity relates to psychological and health outcomes. For example, work by Bravo *et al.* (2016) found evidence that membership to different groups of religiosity is differentially associated with psychological and health outcomes. Specifically, high and low religiosity groups differed in levels of rumination, with the more religious individuals being less likely to ruminate, and lower levels of alcohol consumption were found in intrinsically compared with extrinsically motivated religious groups. Despite the existence of several studies that use latent class analyses to examine religiosity ( Bravo *et al.*, 2016; Tong & Yang, 2018; Vasilenko & Espinosa-Hernández, 2019), there is still work to be done to understand how religious beliefs and behaviours cohere in an individual to form their personal religiosity. Previous studies predominantly examined adolescent religiosity – which can be a time of significant religious change ( Desmond *et al.*, 2010; McNamara Barry *et al.*, 2010; Paloutzian & Park, 2005). This suggests that the latent classes found in studies of adolescents may not be reflective of adult populations – a possibility that has been neglected by the existing research. These studies also exclusively examine US samples, which have been shown to differ from many other countries in their religious beliefs ( Fahmy, 2021), and in outcomes associated with religiosity ( Zimmer *et al.*, 2019).

The study also aims to examine associations between patterns of religiosity and a range of socioeconomic indicators. The intention of the present study is to provide a set of latent classes for use in examining prospective associations, in outcomes relating to both parent and offspring.

## Methods

### Participants

 The Avon Longitudinal Study of Parents and Children (ALSPAC) was established to understand how genetic and environmental characteristics influence health and development in mothers, partners, and children. All pregnant women resident in a defined area in the Southwest of England, with an expected date of delivery between 1st April 1991 and 31st December 1992, were eligible and 13 761 women (contributing 13 867 pregnancies). These mothers and their partners have been followed over the last 30 years and have completed a variety of questionnaires concerning their demographics, physiological and genetic data, life events, physical, and psychological characteristics. For more information, see Boyd *et al.* (2013); Fraser *et al.* (2013). Partners were not formally recruited for the majority of ALSPAC’s existence, instead relying upon mothers giving their partners a questionnaire to complete. For this reason, the early waves of ALSPAC have partner samples that fluctuate. Of special relevance to this study, ALSPAC includes a number of measures of religious belief and behaviour. It also contains a number of sociodemographic variables, which allows us to examine whether there are significant demographic differences in the latent classes that emerge. 

A fully searchable data dictionary and variable search tool exist on the study website. This tool provides an easy identification tool for researchers looking for variables relating to a specific topic over the length of the study.

We conducted our analyses on the mothers and partners from the ALSPAC cohort. After removing participants who had withdrawn consent or not taken part in the wave of interest (1332 mothers and 47 partners), our sample consisted of 12429 mothers and 9953 partners at baseline. It should be noted that a number of factors are associated with participation in ALSPAC, such as socioeconomic, health and lifestyle factors ( Cornish *et al.,* 2021; Fernández-Sanlés *et al.,* 2021), which may be sources of bias in this sample.

Compared to the partners’ cohort, the mothers’ cohort was younger and had a higher proportion with high school qualifications, but a smaller proportion with a degree. A smaller proportion of the mothers’ compared with the partners’ cohort worked in a manual occupation or had financial problems or hardship. See Table 1 for a summary of the sample.

**Table 1.** Mean age and distribution of sociodemographic factors in the mothers’ and partners’ ALSPAC cohorts.

Variable	Mothers			Partners		
		Response frequency	Mean (SD)		Response frequency	Mean (SD)
Age at baseline			28.3 (4.8)			30.9 (5.7)
Education	CSE/vocational/none O-level/A-level Degree	28.9% 57.9% 13.2%		CSE/vocational/none O-level/A-level Degree	30.0% 50.5% 19.5%	
Car access	Yes No	90.2% 9.8%				
Social class	Non-manual Manual	78.2% 21.8%		Non-manual Manual	54.6% 45.4%	
Housing status	Rented Bought	18.6% 81.4%		Rented Bought	16.1% 83.9%	
Financial problems	Yes No	88.1% 11.9%		Yes No	86.3% 13.7%	
Financial hardship	Yes No	25.5% 74.5%		Yes No	24.9% 75.1%	

### Ethics and consent

Ethical approval for the study was obtained from the ALSPAC Ethics and Law Committee (ALEC; IRB00003312) and the Local Research Ethics Committees. Informed consent for the use of data collected via questionnaires and clinics was obtained from participants following the recommendations of the ALSPAC Ethics and Law Committee at the time ( Birmingham & Golding, 2018). Detailed information on the ways in which confidentiality of the cohort is maintained may be found on the ALSPAC study website.

### Measures

***Religiosity.*** Mothers and partners completed questionnaires on religious beliefs and behaviours, of which, 89% were administered during pregnancy; 11% post-delivery. We elected to collapse response options for several items, due to them functioning poorly for Atheists and Agnostics. See Table 2 for details of the retained items and recoded responses. 

**Table 2.** Original and recoded response options for religiosity items.

Measure	Original response options	Recoded response options	Frequency Mothers	Frequency Partners
1) Do you believe in God or in some divine power?	**Yes** **Not sure** **No**	**Yes** **Not sure** **No**	**49.8%** **35.3%** **14.9%**	**37.0%** **34.4%** **28.6%**
2) Do you feel that God (or some divine power) has helped you at any time?	**Yes** **Not sure** **No**	**Yes ** **Not sure** **No**	**33.9% ** **37.9%** **28.3%**	**25.3%** **32.3%** **42.4%**
3) Would you appeal to God for help if you were in trouble?	**Yes** **Not sure** **No**	**Yes** **Not sure** **No**	**46.6%** **31.4%** **22.1%**	**36.1%** **27.5%** **36.4%**
4) How long have you had this particular faith?	**All my life ^1^** **More than 5 years ^1^** **3–5 years ^2^** **1–2 years ^2^** **Less than a year ^2^** **No response ^1^**	**Long-time (non)believer ^1^** **Recent convert ^2^**	**96.9%** **3.1%**	**96.7%** **3.3%**
5) Do you go to a place of worship?	**Yes, at least once a week ^1^** **Yes, at least once a month ^1^** **Yes, at least once a year ^2^** **Not at all ^2^** **No response ^2^**	**Religious attendance ^1^** **Occasional attendance ^2^**	**14.3%** **85.7%**	**10.4% ** **89.6%**
6) Do you obtain help and support from leaders [other members] of your [other] religious group?	**Yes ^1^** **No ^2^** **No response ^2^**	**Yes ^1^** **No ^2^**	**11.3%** **88.7%**	**8.0%** **92.0%**

**Note.** Superscript indicates recoding pattern.

### Sociodemographic variables

The sociodemographic variables included participants’ (mothers and partners) age (at baseline) and a range of indicators of socioeconomic position including educational attainment, car access, social class, housing status, financial problems, and financial hardship.

Mother and partner education was defined as 1; *Certificate of secondary school education/vocational/none*, 2; *A-level or O-level*, 3; *Degree*.

Occupational social class was assessed based on the mother or partner’s occupational social class, which was derived using the 1991 British Office of Population and Census Statistics (OPCS) classification and dichotomized into nonmanual (professional, managerial or skilled professions) and manual (partly or unskilled occupations) (for more information, see ( *The National Statistics Socio-Economic Classification (NS-SEC) - Office for National Statistics*, 2021).

We used four binary indicators of financial status: financial hardship (whether they had struggled to afford food, clothing, heating, accommodation, or things for their baby), major financial problems (whether they felt they had experienced major financial problems), home ownership (living in owner/occupier versus rented accommodation) and car access.

### Statistical analyses

We conducted a latent class analysis on our selected religious belief items. The latent class analysis was carried out using R ( R Core Team, 2021) and the poLCA package ( Linzer & Lewis, 2011). We also used multinomial logistic regression, using sociodemographic variables as exposures and latent class as the outcome variable to examine differences between the latent classes. This analysis was carried out using Mplus (Version 8.7).

## Results

### Model choice

Our criteria for model selection were based upon the Bayesian Information Criteria (BIC) ( Nylund *et al.*, 2007), the entropy values for each class, and the interpretability of each class extraction ( Marsh *et al.*, 2009; Meeus *et al.*, 2011; Schreiber, 2017). These criteria were also used to select a model for the partners of the mothers. The BIC for mothers indicated that a 5-class solution should be retained and a 6-class solution in the partners. However, these solutions produced classes consisting of approximately less than 2% of the sample, which was interpreted as a sign of overextraction. This led to us dropping the 5-class solution for mothers, 6-class solution for partners, and retaining a 4-class solution for both mothers and partners. See the supplementary materials under *Extended data* for model fit tables ( Halstead, 2022).

### Class descriptions

These classes conformed to a broad continuum of religiosity. Based on the conditional probabilities, we assigned subjective labels that we felt provided a broad summary of the class. We named Class 1 the ‘Highly Religious’ class due to their consistent endorsement of religious beliefs, high level of church attendance, and high likelihood to obtain help and support from religious individuals. We named Class 2 the ‘Moderately Religious’ group as they shared many of the characteristics of Class 1 but were highly unlikely to visit church regularly. We named Class 3 ‘Agnostics’ as they expressed an uncertainty about the existence of God and were also uncertain about whether they would ask for help from God if they were in trouble or whether God had helped them previously. We named Class 4 ‘Atheists’ due to their strong disbelief in the existence of God, and general disagreement with statements about appealing to God for help or whether they had received help from God previously. All classes were highly likely to indicate that they were long time believers (or non-believers in the case of Atheists)– barring a slightly increased likelihood to be a recent convert in Class 1 (Highly Religious).

When comparing mother and partner classes, there was a higher proportion of partners in the Atheist class compared to mother classes, and a decrease in the highly and moderately religious classes. For full details see Table 3.

**Table 3.** Summary table of each class and their descriptions based on conditional probabilities.

Class name	Class description
High religious	Consistent endorsement of religious beliefs, regular church attendance, and high likelihood to obtain help and support from religious individuals.
Moderately Religious	Share many of the characteristics of the highly religious but were highly unlikely to visit church regularly or receive help from religious sources.
Agnostics	Express an uncertainty about the existence of God and were also uncertain about whether they would ask for help from God if they were in trouble or whether God had helped them previously.
Atheists	Strong disbelief in the existence of God, and general disagreement with statements about appealing to God for help or whether they had received help from God previously

### Bias adjusted latent class solutions

We then examined whether there was an association between partner participation in ALSPAC and latent class membership, in order to examine the possibility that religious class created a selection bias in partner participation. A bias-corrected three step approach (as discussed in Asparouhov & Muthén, 2014; Heron *et al.*, 2015) was used to control for the possibility of bias within the sample. This approach involves estimating latent classes, obtaining a latent class variable based on the modal posterior class membership probability, which is then used as a latent class indicator variable in order to account for the measurement error. During the final step we also regressed modal class assignment on distal variables to control for misclassification in the earlier steps and examined whether they provided a better class resolution. In this step, partner missingness was included to account for the possibility of religiosity, and mother-partner religious concordance in the families relating to participation in ALSPAC ( Hur, 2007; McClendon, 2016). Including partner missingness in the model estimation process resulted in no change to our class solutions. For full details see Table 4.

**Table 4.** Conditional probabilities and class shares for mothers and partners.

	Mothers				Partners			
	Class 1:	Class 2:	Class 3:	Class 4:	Class 1:	Class 2:	Class 3:	Class 4:
**Class shares**	.11	.30	.38	.21	.08	.23	.34	.35
**Do you believe in God or in some divine power?**								
Yes	1.00	.93	.24	.06	1.00	.89	.19	.05
Not sure	.00	.07	.75	.24	.00	.10	.78	.16
No	.00	.00	.01	.70	.00	.00	.03	.79
**Do you feel that God (or some divine power) ** **has helped you at any time?**								
Yes	.95	.73	.02	.00	.95	.74	.02	.00
Not sure	.05	.25	.78	.01	.05	.24	.77	.01
No	.00	.02	.20	.99	.00	.03	.22	.99
**Would you appeal to God for help if you were in ** **trouble?**								
Yes	.98	.89	.22	.01	.99	.83	.24	.02
Not sure	.01	.09	.71	.08	.01	.11	.65	.07
No	.00	.01	.08	.91	.00	.05	.10	.91
**How long have you had this particular faith?**								
Long-time (non)believer	.89	.96	1.00	.97	.85	.96	1.00	.97
Recent convert	.11	.04	.00	.03	.15	.04	.00	.03
**Do you go to a place of worship?**								
Religious attendance	.87	.09	.03	.01	.92	.06	.03	.01
Occasional attendance	.13	.91	.97	.99	.08	.94	.97	.99
**Do you obtain help and support from leaders ** **[other members] of your [other] religious ** **group?**								
Yes	.84	.03	.02	.00	.80	.03	.02	.01
No	.16	.97	.98	1.00	.20	.97	.98	.99

### Associations between parental latent classes

We next examined the possibility of assortative mating in the mother-partner dyads (i.e., a preference for a partner with the same pattern of religious (non)belief as themselves). This was done using Mplus (Version 8.7), firstly by generating latent classes for both mothers and partners while keeping item responses between mothers and partners invariant. The logits for most likely class membership from this step were then used to generate classes separately. Finally, we regressed the mother’s class on the partner’s class. We found that the Highly Religious and Atheist classes were most likely to have a partner with the same class as themselves. While there was a preference for partners in the same latent class as themselves, the Agnostic and Mildly Religious classes were more likely to have a partner from a different latent class. See Table 5 for more details.

**Table 5.** Associations between parental latent class.

			Partner data		
		Highly Religious	Moderately Religious	Agnostic	Atheist
	Highly religious	0.599	0.136	0.142	0.123
**Mother** ** data**	Moderately religious	0.008	0.373	0.367	0.251
	Agnostic	0.007	0.191	0.426	0.376
	Atheist	0.008	0.103	0.283	0.606

### Associations between the sociodemographic indicators and the latent classes of religion in mothers and partners

The Highly Religious classes possessed a higher proportion of individuals with degree level education, non-manual social class, and a lower proportion who reported financial hardship. The Atheist classes had the highest proportion of individuals with CSE/Vocational/No qualifications. Overall, the mothers’ classes had a higher proportion of manual workers, a lower level of education, and were less likely to report financial problems, compared to the partners classes. See Table 6 for more details. All these associations were unadjusted for confounders.

**Table 6.** Unadjusted associations between the sociodemographic indicators by parental religious class.

	Social class (Mother)	Social class (Partner)		Financial problems (Mother)	Financial problems (Partner)	Financial hardship (Mother)	Financial hardship (Partner)		Age (Mother)	Age (Partner)		Education (Mother)	Education (Partner)
	Proportion (SE)	Proportion (SE)		Proportion (SE)	Proportion (SE)	Proportion (SE)	Proportion (SE)		Proportion (SE)	Proportion (SE)		Proportion (SE)	Proportion (SE)
**Highly** ** religious**													
Non-manual class	. 920 (.013)	.884 (.028)	Yes	. 112 (.011)	. 127 (.019)	.207 (.014)	. 195 (.026)	Less than 25	.075 (.011)	. 030 (.015)	CSE/Vocational/ None	. 082 (.012)	. 066 (.022)
Manual class	. 080 (.013)	.116 (.028)	No	. 888 (.011)	. 873 (.019)	.793 (.014)	. 805 (.026)	25–30	.414 (.016)	. 375 (.031)	O-Level/A-level	. 615 (.016)	. 407 (.028)
								31+	.511 (.017)	.595 (.031)	Degree	.303 (.014)	. 527 (.029)
**Moderately** ** religious**													
Manual class	. 564 (.008)	.808 (.013)	Yes	. 120 (.006)	. 144 (.008)	237 (.008)	.280 (.013)	Less than 25	.159 (.007)	.089 (.008)	CSE/Vocational/ None	. 267 (.008)	. 300 (.010)
Non-manual class	. 436 (.008)	.192 (.013)	No	. 880 (.006)	. 856 (.008)	.880 (.008)	.720 (.013)	25–30	.506 (.009)	.410 (.014)	O-Level/A-level	. 615 (.009)	. 542 (.011)
								31+	.336 (.009)	.501 (.014)	Degree	. 118 (.006)	. 158 (.008)
**Agnostic**													
Manual class	. 492 (.009)	.739 (.007)	Yes	. 113 (.006)	. 140 (.007)	.265 (.008)	.236 (.010)	Less than 25	.224 (.008)	.093 (.007)	CSE/Vocational/ None	.339 (.009)	. 317 (.009)
Non-manual class	. 508 (.009)	.261 (.012)	No	. 887 (.006)	. 860 (.007)	.735 (.008)	.764 (.010)	25–30	.506 (.008)	.423 (.012)	O-Level/A-level	.576 (.009)	. 520 (.010)
								31+	.270 (.008)	.484 (.012)	Degree	.085 (.005)	. 163 (.007)
**Atheists**													
Non-manual class	. 522 (.010)	.750 (.009)	Yes	. 129 (.007)	. 132 (.006)	.296 (.010)	.250 (.009)	Less than 25	.293 (.010)	.112 (.006)	CSE/Vocational/ None	. 355 (.010)	. 324 (.008)
Manual class	. 478 (.010)	.250 (.009)	No	. 871 (.007)	. 868 (.006)	.704 (.010)	.750 (.009)	25–30	.431 (.011)	.410 (.010)	O-Level/A-level	. 505 (.010)	. 483 (.008)
								31+	.276 (.010)	.478 (.010)	Degree	.140 (.007)	. 194 (.007)

## Discussion

 The results of our analysis show that religious beliefs in the ALSPAC mothers and partners cohorts cohere into four heterogeneous classes that conformed to a broad continuum of religiosity. These classes were labelled as the Highly Religious, Moderately Religious, Agnostics, and Atheists. Of these classes, the Highly Religious class was associated with a higher level of socioeconomic position and educational attainment than the other classes.

The religious beliefs of both the mother and partner samples was adequately captured with the same 4-class solution. These class solutions possessed similar conditional response probabilities but varied in the proportion of the sample that belonged to each class. Specifically, a higher proportion of partners were members of the Atheist and Agnostic groups than the mothers. This finding aligns with the literature that examines sex differences in religious beliefs, which notes that women score higher on measures of religiosity ( Feltey & Poloma, 1991; Levin *et al.*, 1994; Schnabel, 2015).

### Strengths and limitations

A key strength of the study is that it is based on data from a large cohort of adults. With an approximate sample size of 22,000 this is the largest latent class analysis of religiosity to date. The use of a UK sample provides a novel insight into patterns of religiosity, given that previous research was based almost exclusively on US samples. Furthermore, we took methodological steps to account for measurement issues in the questionnaires, such as using the bias adjusted three-step approach, as well as performing an examination of sources of bias within the sample, which has not been accounted for in previous studies.

There are also limitations associated with this study that should be considered when interpreting the findings. The ALSPAC cohort is homogenous in terms of ethnicity (being predominately white). Of those who reported a religious faith, the majority identified as Christian. This prevents the present study from being able to draw conclusions about whether religious latent classes cohere differently in other ethnicities and faiths. The items used in the early waves of the mothers’ and partners’ cohorts in ALSPAC appear to function poorly for those who are Agnostic or Atheist. This issue is apparent from the large number of missing responses (from the duration of faith, religious attendance, and help from members of a faith items) in those who indicated they were members of these groups. While we took steps in the present study to minimise the number of missing responses, by recoding a number of patterns of missingness, it is important that future data collection considers the differential item functioning of their religiosity questions. Furthermore, there is the possibility that being pregnant may cause changes in a mother’s religiosity or spirituality, which may bias their responses at the time period examined in this study.

### Comparison with previous research

The existence of a consistently, highly religious class is in line with the findings of previous latent class analyses ( Bravo *et al.*, 2016; Tong & Yang, 2018; Vasilenko & Espinosa-Hernández, 2019). Compared to these studies, the existence of large Agnostic and Atheist classes in our sample is novel, which may be attributable the focus in previous research on samples with at least a modest level of religiosity, rather than the general public.

The time at which these questions were asked, as well as the age of the participants provides an interesting snapshot of religiosity from 1991-2 in the UK. More recent research in the US has identified a proportion of individuals who could be considered ‘spiritual but not religious’, or indicate that they are non-religious, but still exhibit religious behaviours such as attending church ( Tong & Yang, 2018). In our sample, the Moderately religious class may be a UK equivalent to a spiritual but not religious class, in which they identify as believing in god, but do not perform behaviours associated with being religious. Alternatively, this class may just be an expression of diminished religious belief, that may make them more prone to transitioning to the Agnostic and Atheist classes over time ( Marshall & Olson, 2018; Streib, 2008).

## Conclusions

This study provides an insight into patterns of religiosity in a large UK cohort. Our results indicate the presence of four qualitatively different classes of religiosity which go beyond the traditional assumptions of religiosity existing on a simple continuum. Furthermore, this study highlights the complexity of examining religiosity and the need for diverse approaches to examining this fundamental human experience. These classes could be used to examine prospective relationships between religiosity and psychosocial and health outcomes in mothers, partners and children from the ALSPAC cohort.

## Data Availability

ALSPAC data access is through a system of managed open access. The steps below highlight how to apply for access to the data included in this research article and all other ALSPAC data. The datasets presented in this article are linked to ALSPAC project number B3892, please quote this project number during your application. The ALSPAC variable codes highlighted in the dataset descriptions can be used to specify required variables. 1. Please read the ALSPAC access policy which describes the process of accessing the data and samples in detail, and outlines the costs associated with doing so. 2. You may also find it useful to browse our fully searchable research proposals database, which lists all research projects that have been approved since April 2011. 3. Please submit your research proposal for consideration by the ALSPAC Executive Committee. You will receive a response within 10 working days to advise you whether your proposal has been approved. If you have any questions about accessing data, please email alspac-data@bristol.ac.uk. The study website also contains details of all the data that is available through a fully searchable data dictionary. OSF: Supplementary Materials. [ 10.17605/OSF.IO/Z639X]. ( Halstead, 2022) This project contains the following extended data: A Latent Class Approach to Religiosity Using a Large UK supplementary materials.docx Data are available under the terms of the Creative Commons Attribution 4.0 International license (CC-BY 4.0).
