# Supplementary material for: Identifying patterns of religiosity in adults from a large UK cohort using latent class analysis
Source: Wellcome Open Res. 2026 Jan 27;7:192. Originally published 2022 Jul 18. [Version 3] doi: 10.12688/wellcomeopenres.17969.3 (PMC12936478; doi:10.12688/wellcomeopenres.17969.3)
Supplement: Peer review history

# Reviewer report: Reviewer response for version 3

**DOI:** 10.21956/wellcomeopenres.28193.r145684

**Published:** 2026-02-25

**Authors:** Telli Davoodi

**Affiliations:** 1 Wheelock College of Education & Human Development, Boston University, Boston, USA; 2 Princeton University, Princeton, USA

This version looks good to me. Nice work!

Is the work clearly and accurately presented and does it cite the current literature?

Partly

If applicable, is the statistical analysis and its interpretation appropriate?

Partly

Are all the source data underlying the results available to ensure full reproducibility?

Yes

Is the study design appropriate and is the work technically sound?

Yes

Are the conclusions drawn adequately supported by the results?

Partly

Are sufficient details of methods and analysis provided to allow replication by others?

No

Reviewer Expertise:

Social and religious cognition, taking cross-cultural and developmental approaches.

I confirm that I have read this submission and believe that I have an appropriate level of expertise to confirm that it is of an acceptable scientific standard.

# Reviewer report: Reviewer response for version 3

**DOI:** 10.21956/wellcomeopenres.28193.r145683

**Published:** 2026-02-04

**Authors:** Annibale Cois

**Affiliations:** 1 School of Public Health, University of Cape Town, Cape Town, South Africa; 2 South African Medical Research Council, Cape Town, South Africa

The authors have addressed my concerns adequately, and the new version of the manuscript includes clearer and more detailed explanations of the methodological choices. 

 This will allow the readers to conduct a more comprehensive evaluation of the significance and limitations of the findings.

 A minor comment: 

 - I suggest clarifying that the 3-step approach's main aim is to include in the modelling the uncertainty in the class attribution (especially when entropy is low)

Is the work clearly and accurately presented and does it cite the current literature?

Partly

If applicable, is the statistical analysis and its interpretation appropriate?

Partly

Are all the source data underlying the results available to ensure full reproducibility?

Yes

Is the study design appropriate and is the work technically sound?

Partly

Are the conclusions drawn adequately supported by the results?

Partly

Are sufficient details of methods and analysis provided to allow replication by others?

No

Reviewer Expertise:

Statistical modelling, Structural Equation Modelling, Survey data, Epidemiology of non-communciabel diseases

I confirm that I have read this submission and believe that I have an appropriate level of expertise to confirm that it is of an acceptable scientific standard.

# Reviewer report: Reviewer response for version 2

**DOI:** 10.21956/wellcomeopenres.26540.r138649

**Published:** 2025-11-26

**Authors:** Annibale Cois

**Affiliations:** 1 School of Public Health, University of Cape Town, Cape Town, South Africa; 2 South African Medical Research Council, Cape Town, South Africa

The study described in the article employs a latent class analysis (LCA) approach to identify heterogeneous patterns of religiosity and some of their socioeconomic correlates among participants (mothers and their partners) in a large cohort study established in the early 1990s in the Southwest of England. The analyses reported in the article are cross-sectional and refer to the data gathered during the baseline collection.

 From a methodological point of view (the only aspect of the study I am discussing in this review, as my field of expertise does not include religious studies), the overall approach appears coherent with the objectives of the study of capturing the multidimensional nature of the construct of interest and providing a measurement tool for the analysis of prospective association with outcomes captured at subsequent points in time among the cohort participants.

 However, I have reservations regarding the actual implementation of the LCA analysis and the lack of clarity in reporting methodological details and results. Below my specific comments.

1. Item functioning

The Authors state that some items “functioned poorly” among some classes of participants, but a formal definition of poor functioning and clear assessment criteria are lacking. If the Authors are referring to the problem of differential functioning of some items across latent classes, some form of quantification of this effect (before and after the collapsing of the response options) should be provided.

1. Missing data

The Authors cite among the limitations a “ *large number of missing responses*” especially among participants in some latent classes, but it is not clear how the missing data were treated during the analyses. Did the Authors excluded cases with missing values for the LCA analyses (listwise deletion, the default in poLCA) or did they use different strategies (e.g. FILM estimator, also available in poLCA, multiple imputation, sensitivity analyses?) Also, it is not clear what the Authors mean by “ *minimise the number of missing responses, by recoding a number of patterns of missingness*” (limitations section) or “ *pattern missingness was included*” (in the Bias adjusted latent class solutions).

 I suggest that The Author include in the Methods section details regarding the treatment of missing data and the underlying rationale. A summary table with the proportion of missing data across variables would also contribute to understanding the extent of the risk of bias. 

1. Reporting

I found the reporting of methodological details sometimes confusing and, in general, insufficient for a critical evaluation of the analyses. Some examples below:

- the Methods section indicates that the relationships between sociodemographic variables and latent class (membership) are analysed by using multinomial logistic regression, which estimates regression coefficients/odds ratios. Table 6, however, looks more like a cross-tabulation table with proportions: how are these proportions (and their standard errors) calculated from the outputs of the multinomial models?
- The description of the “ *Bias adjusted class solutions*” is very unclear. As reported in the cited article by Heron et Al., the denomination of “three-step approach” refers to a set of different techniques.  It is not clear to me if the specific three-step approach used takes into account the uncertainty in the modal class assignment (quite large in this case: see comment on class separation below).

Also, which distal variables were considered, and how does their inclusion control for misclassification?

 What does it mean “ *including partner missigness*”? The results in Table 4 do/do not include that variable?

- In the “ *Associations between parental latent classes*” section, I assume that the statement “ *keeping item responses between mothers and partners invariant*” refers to keeping factor *loadings* invariant. The Authors should explain the reason of keeping those values constant (and why only in this specific analysis). Also, the analysis is described as a regression, but Table 5 reports proportions across classes. How are these proportions calculated?
-  In describing the sample and in the results section, the Authors comment regarding differences and similarities between participants (mother vs. partners) and between latent classes. A note in the methods section should specify how these differences and similarities are assessed from a statistical point of view (type of test, level of significance).

I suggest the author revise the whole description of the methods, with the objectives of producing unambiguous descriptions, including variables used, estimation methods, etc. When regression analysis is used, the model outputs (estimated regression coefficients and their standard errors)  should be made available. 

1. Class separation

The values of Entropy for the chosen 4-classes solution in Table S1 suggest very poor class separation, especially among partners. Even solutions with reduced number of classes do not seem to improve significantly the picture.

 With the levels of entropy, an established literature indicates that using the “most likely class membership”  variable for further analyses as if it were an observed variable (without considering its uncertainty) is problematic.  The same article by Haron et al (2015) cited in the Manuscript concludes that ignoring class uncertainty in analysing the relationships between class membership and predictors is a source of bias and misestimation (often underestimation) of standard errors. In this perspective, the results in Table 6 need to be considered with extreme caution, especially (but not only) for the partner’s subsamples.

 Have the authors considered using a single-step approach to estimate the latent classes together with their relationships with the demographic variables? Or a three-step approach with inclusion of class assignment uncertainty? With those levels of entropy, I don’t think that the problem can be overlooked.

 In any case, I suggest the Authors to report in the additional material a more exhaustive description of the results of the LCA analysis. E,g, the whole confusion matrix could allow the readers to better understand if the problem of poor separation is specific to some classes or more general. The results or limitations section should also be used to make the readers aware of the potential bias arising from poor class separation.

 Minor comment2

1. Table1: misplaced rows (e.g. Education in mothers; labels of Education
2. All tables: revise to ensure unambiguous description of their content.

Is the work clearly and accurately presented and does it cite the current literature?

Partly

If applicable, is the statistical analysis and its interpretation appropriate?

Partly

Are all the source data underlying the results available to ensure full reproducibility?

Yes

Is the study design appropriate and is the work technically sound?

Partly

Are the conclusions drawn adequately supported by the results?

Partly

Are sufficient details of methods and analysis provided to allow replication by others?

No

Reviewer Expertise:

Statistical modelling, Structural Equation Modelling, Survey data, Epidemiology of non-communciabel diseases

I confirm that I have read this submission and believe that I have an appropriate level of expertise to confirm that it is of an acceptable scientific standard, however I have significant reservations, as outlined above.

# Response

**Published:** 2025-12-09

**Authors:** Isaac Halstead

**Affiliations:** University of Bristol, UK

**Thank you to our reviewers who have taken the time to review this revised version of the manuscript, especially given the length of time this paper has been in processing by WOR. We have made a number of changes to the manuscript, which you will see below. Our responses are in bold, and excerpts from the manuscript are italicised.**

 Reviewer 1: I think the revisions have made the writing clearer and some of the researcher decisions are not more transparent. I think this revised version adds to the literature and can inform future decisions about measures for religiosity. I would like to see the authors give more thought to the age of this data as an important limitation. Especially, when it comes to religiosity, the global landscape is changing rapidly, and it is not clear how much data almost 35 years ago can still tell us about patterns today.

**Response:** **Thank you for this feedback. We have now made it clearer in the discussion section the limitations and value of the measures from this timepoint:**

*We should also acknowledge that parental religiosity is taken from almost 35 years ago. The parent’s religiosity is likely to have changed in the intervening years (although there is evidence for a degree of stability across multiple timepoints (Major-Smith et al., 2023)), as well as society’s relationship with religiosity (Duffy et al., 2023). However, these parental data are still useful, as it allows us to examine the prospective associations with their offspring’s outcomes, and the role religiosity may play from childhood, into adolescence, and adulthood (e.g. Halstead et al., 2023, 2024a, 2024b).*

 Reviewer 2: The study described in the article employs a latent class analysis (LCA) approach to identify heterogeneous patterns of religiosity and some of their socioeconomic correlates among participants (mothers and their partners) in a large cohort study established in the early 1990s in the Southwest of England. The analyses reported in the article are cross-sectional and refer to the data gathered during the baseline collection.

 From a methodological point of view (the only aspect of the study I am discussing in this review, as my field of expertise does not include religious studies), the overall approach appears coherent with the objectives of the study of capturing the multidimensional nature of the construct of interest and providing a measurement tool for the analysis of prospective association with outcomes captured at subsequent points in time among the cohort participants.

 However, I have reservations regarding the actual implementation of the LCA analysis and the lack of clarity in reporting methodological details and results. Below my specific comments.

1. Item functioning

The Authors state that some items “functioned poorly” among some classes of participants, but a formal definition of poor functioning and clear assessment criteria are lacking. If the Authors are referring to the problem of differential functioning of some items across latent classes, some form of quantification of this effect (before and after the collapsing of the response options) should be provided.

**Thank you for pointing this out. Item functioning is probably not the best way to describe what we meant, so we have amended the paragraph to specify the issue:**

*We elected to collapse response options for several items, as they may have been prone to misreporting. For example, not attending at all or at least once a year may be inaccurately reported by participants, depending on whether they consider weddings/funerals as church attendance. This approach also aligns with the treatment of attendance in the literature, where regular and occasional/no attendance is often compared* *(e.g. Bulbulia et al., 2024)*

1. Missing data

The Authors cite among the limitations a “ *large number of missing responses*” especially among participants in some latent classes, but it is not clear how the missing data were treated during the analyses. Did the Authors excluded cases with missing values for the LCA analyses (listwise deletion, the default in poLCA) or did they use different strategies (e.g. FILM estimator, also available in poLCA, multiple imputation, sensitivity analyses?) Also, it is not clear what the Authors mean by “ *minimise the number of missing responses, by recoding a number of patterns of missingness*” (limitations section) or “ *pattern missingness was included*” (in the Bias adjusted latent class solutions).

**Thank you for the feedback, partially incomplete data within the set of latent class indicators was addressed through the use of FIML.  Anyone with 1 or more responses to these data was included in the estimation of the measurement model. We have now clarified what we did when discussing the limitations:**

*There are also limitations associated with this study that should be considered when interpreting the findings. The ALSPAC cohort is homogenous in terms of ethnicity (being predominately white). Of those who reported a religious faith, the majority identified as Christian. This prevents the present study from being able to draw conclusions about whether religious latent classes cohere differently in other ethnicities and faiths. The items used in the early waves of the mothers’ and partners’ cohorts in ALSPAC appear to function poorly for those who are Agnostic or Atheist. This issue is apparent from the large number of people who chose non-response (from the duration of faith, religious attendance, and help from members of a faith items) in those who indicated they were members of these groups. While we took steps in the present study to minimise the number of missing responses, by recoding a number of patterns of missingness for example, if someone indicated they did not believe in god or a divine power, and did not respond to the religious support, church attendance items, then they were recoded as occasionally attending church, and not having received support. It is important that future data collection considers the differential item functioning of their religiosity questions. Furthermore, there is the possibility that being pregnant may cause changes in a mother’s religiosity or spirituality, which may bias their responses at the time period examined in this study.*  

**We have also made it clearer in the methods how the remaining missingness was treated when conducting the latent class analysis:**

*After recoding the data within certain patterns of missingness, partially incomplete data within the set of latent class indicators was addressed through the use of FIML. anyone with one or more response to these data was included in the estimation of the measurement model.* I suggest that The Author include in the Methods section details regarding the treatment of missing data and the underlying rationale. A summary table with the proportion of missing data across variables would also contribute to understanding the extent of the risk of bias. 

**Thank you for this feedback, we have also amended the methods section to make it clear what was done to reduce missingness in some of the items:**

*We also examined non-response in the duration of faith, attendance, and religious support items, and whether this was among non-believers, in the event these items were not functioning properly for them. We then recoded the non-believers who had not responded to the duration of faith, attendance, and religious support items, as being long time believers, occasional attenders, and not receiving help from religious groups. This was done on the assumption that they were unsure of how to respond to these items that pre-suppose a faith/belief given their previous expression of non-belief.*

1. Reporting

I found the reporting of methodological details sometimes confusing and, in general, insufficient for a critical evaluation of the analyses. Some examples below:

- the Methods section indicates that the relationships between sociodemographic variables and latent class (membership) are analysed by using multinomial logistic regression, which estimates regression coefficients/odds ratios. Table 6, however, looks more like a cross-tabulation table with proportions: how are these proportions (and their standard errors) calculated from the outputs of the multinomial models?
- The description of the “ *Bias adjusted class solutions*” is very unclear. As reported in the cited article by Heron et Al., the denomination of “three-step approach” refers to a set of different techniques.  It is not clear to me if the specific three-step approach used takes into account the uncertainty in the modal class assignment (quite large in this case: see comment on class separation below).

Also, which distal variables were considered, and how does their inclusion control for misclassification?

 What does it mean “ *including partner missigness*”? The results in Table 4 do/do not include that variable?

**Thank you for pointing this out, we have now amended the paragraph to be clearer:**

*We then examined whether there was an association between partner participation in ALSPAC and latent class membership, in order to examine the possibility that religious class created a selection bias in partner participation. A bias-corrected three step approach (called modal ML, as discussed in* *Asparouhov & Muthén, 2014* *;* *Heron et al., 2015* *) was used to control for the possibility of bias within the sample. This approach involves estimating latent classes, obtaining a latent class variable based on the modal posterior class membership probability, which is then used as a latent class indicator variable in order to account for the uncertainty in the modal class assignment. The final entropy for the maternal and paternal latent classes were .982 and .843 respectively. During the final step we also regressed modal class assignment on distal variables to control for misclassification in the earlier steps and examined whether they provided a better class resolution. Our chosen distal variables were partner absence, to account for the possibility of religiosity, and mother-partner religious concordance in the families relating to participation in ALSPAC (* *Hur, 2007* *;* *McClendon, 2016* *). Including partner missingness in the model estimation process resulted in no change to our class solutions. For full details see* *Table 4* *.*

 In the “Associations between parental latent classes” section, I assume that the statement “keeping item responses between mothers and partners invariant” refers to keeping factor loadings invariant. The Authors should explain the reason of keeping those values constant (and why only in this specific analysis). Also, the analysis is described as a regression, but Table 5 reports proportions across classes. How are these proportions calculated?

**Thank you for your suggestions, we have amended the paragraph accordingly:**

*We next examined the possibility of assortative mating in the mother-partner dyads (i.e., a preference for a partner with the same pattern of religious (non)belief as themselves). This was done using Mplus (Version 8.7), firstly by generating latent classes for both mothers and partners while keeping factor loadings between mothers and partners invariant. This was done to preserve the 4-class latent class structure between them, to allow for better comparisons between maternal/paternal classes. The logits for most likely class membership from this step were then used to generate classes separately. Finally, we regressed the mother’s class on the partner’s class. We found that the Highly Religious and Atheist classes were most likely to have a partner with the same class as themselves. While there was a preference for partners in the same latent class as themselves, the Agnostic and Mildly Religious classes were more likely to have a partner from a different latent class. See* *Table 5* *for more details of the estimated proportions of maternal/paternal class concordance*.

-  In describing the sample and in the results section, the Authors comment regarding differences and similarities between participants (mother vs. partners) and between latent classes. A note in the methods section should specify how these differences and similarities are assessed from a statistical point of view (type of test, level of significance).

**Thank you for pointing this out, we have now specified that we did not test to see if maternal/paternal classes significantly differed from one another:**

*The Highly Religious classes possessed a higher proportion of individuals with degree level education, non-manual social class, and a lower proportion who reported financial hardship. The Atheist classes had the highest proportion of individuals with CSE/Vocational/No qualifications. Overall, the mothers’ classes had a higher proportion of manual workers, a lower level of education, and were less likely to report financial problems, compared to the partners classes. See* *Table 6* *for more details. All these associations were unadjusted for confounders and were not formally tested to examine if they significantly differed from one another.* I suggest the author revise the whole description of the methods, with the objectives of producing unambiguous descriptions, including variables used, estimation methods, etc. When regression analysis is used, the model outputs (estimated regression coefficients and their standard errors) should be made available. 

1. Class separation

The values of Entropy for the chosen 4-classes solution in Table S1 suggest very poor class separation, especially among partners. Even solutions with reduced number of classes do not seem to improve significantly the picture. **Thank you for pointing this out, we should have been clearer when describing the table in the supplemental materials that this was for the classes before the 3-step bias adjusted approach was used.**

 With the levels of entropy, an established literature indicates that using the “most likely class membership” variable for further analyses as if it were an observed variable (without considering its uncertainty) is problematic.  The same article by Haron et al (2015) cited in the Manuscript concludes that ignoring class uncertainty in analysing the relationships between class membership and predictors is a source of bias and misestimation (often underestimation) of standard errors. In this perspective, the results in Table 6 need to be considered with extreme caution, especially (but not only) for the partner’s subsamples.

 Have the authors considered using a single-step approach to estimate the latent classes together with their relationships with the demographic variables? Or a three-step approach with inclusion of class assignment uncertainty? With those levels of entropy, I don’t think that the problem can be overlooked.

**As you have suggested, we did carry out a 3-step approach with the inclusion of class assignment uncertainty, but we could have been much clearer in explaining this to the reader.  We have amended the mention of the supplemental materials to say:**

*See the supplementary materials under Extended data for model fit tables before the 3-step bias adjusted approach which incorporated class assignment uncertainty was applied. *  

**We have also addressed this point above:**

*We then examined whether there was an association between partner participation in ALSPAC and latent class membership, in order to examine the possibility that religious class created a selection bias in partner participation. A bias-corrected three step approach (as discussed in* *Asparouhov & Muthén, 2014* *;* *Heron et al., 2015* *) was used to control for the possibility of bias within the sample. This approach involves estimating latent classes, obtaining a latent class variable based on the modal posterior class membership probability, which is then used as a latent class indicator variable in order to account for the uncertainty in the modal class assignment. The final entropy for the maternal and paternal latent classes were .982 and .843 respectively. During the final step we also regressed modal class assignment on distal variables to control for misclassification in the earlier steps and examined whether they provided a better class resolution. Our chosen distal variables were partner absence, to account for the possibility of religiosity, and mother-partner religious concordance in the families relating to participation in ALSPAC (* *Hur, 2007* *;* *McClendon, 2016* *). Including partner absence in the model estimation process resulted in no change to our class solutions. For full details see* *Table 4* *.*  

**We hope that in providing greater clarity in the manuscript that we have shown evidence that we have addressed the misclassification issue raised by this feedback.**  

 In any case, I suggest the Authors to report in the additional material a more exhaustive description of the results of the LCA analysis. E,g, the whole confusion matrix could allow the readers to better understand if the problem of poor separation is specific to some classes or more general. The results or limitations section should also be used to make the readers aware of the potential bias arising from poor class separation.

**Thank you for this feedback. We have now made it clear that after the 3-step bias adjustment approach, the entropy improved significantly (see above text).** Minor comment2

1. Table1: misplaced rows (e.g. Education in mothers; labels of Education
2. All tables: revise to ensure unambiguous description of their content.

**Thank you for taking the time to spot these, we have now amended the table and revised the labels.**

# Reviewer report: Reviewer response for version 2

**DOI:** 10.21956/wellcomeopenres.26540.r131656

**Published:** 2025-09-19

**Authors:** Telli Davoodi

**Affiliations:** 1 Wheelock College of Education & Human Development, Boston University, Boston, USA; 2 Princeton University, Princeton, USA

I think the revisions have made the writing clearer and some of the researcher decisions are not more transparent. I think this revised version adds to the literature and can inform future decisions about measures for religiosity. I would like to see the authors give more thought to the age of this data as an important limitation. Especially, when it comes to religiosity, the global landscape is changing rapidly and it is not clear how much data almost 35 years ago can still tell us about patterns today.

Is the work clearly and accurately presented and does it cite the current literature?

Partly

If applicable, is the statistical analysis and its interpretation appropriate?

Partly

Are all the source data underlying the results available to ensure full reproducibility?

Yes

Is the study design appropriate and is the work technically sound?

Yes

Are the conclusions drawn adequately supported by the results?

Partly

Are sufficient details of methods and analysis provided to allow replication by others?

No

Reviewer Expertise:

Social and religious cognition, taking cross-cultural and developmental approaches.

I confirm that I have read this submission and believe that I have an appropriate level of expertise to confirm that it is of an acceptable scientific standard, however I have significant reservations, as outlined above.

# Reviewer report: Reviewer response for version 1

**DOI:** 10.21956/wellcomeopenres.19915.r84897

**Published:** 2024-08-05

**Authors:** Sarah Wilkins Laflamme

**Affiliations:** 1 University of Waterloo, Waterloo, Ontario, Canada

This article uses Latent Class Analysis (LCA) with UK ALSPAC data from the early 1990s to generate a more comprehensive measure of religiosity than has been seen in some past research. This study makes a modest contribution to the social scientific study of religion. LCA is still rare in the sociology and psychology of religion disciplines, and so an example of the technique in the field is a relevant methodological research note. Some of the study’s findings are also interesting.

 This said, there are a few key issues with this study that make it, in my opinion, not appropriate to be indexed as a full research article. These include the age of the data, the limited measures included in the LCA, and the lack of discussion of why the LCA in this case produces a better-quality measure than simpler techniques such as a summative scale or a Factor Analysis would have done. I detail these three main issues below.

1. Although it is normal to have a time gap between survey data collection and when researchers are able to index findings from these survey data, 30 years is a bit much. The religious landscape has changed a lot in the UK since the early 1990s (when the ALSPAC data was collected). Relationships between religiosity and socioeconomic variables have potentially changed since then, especially with more religiously diverse immigration to the country since the 1990s and nonreligious populations expanding into more demographic groups as nonreligion has become more normalized in UK society and nonreligious populations have grown in size over the past 30 years. There is no reason provided by the authors why this early 1990s ALSPAC data is better than any more recent data coming from the UK for the purposes of their analyses in this study. More recent waves of the British Social Attitudes survey, the European Values Study and the International Social Survey Programme religion module for example all include more demographically representative samples, better geographic coverage of the UK, data collected in the 2010s and 2020s, and multiple measures of religiosity (more religiosity measures than the ALSPAC data in fact). Yes, their sample sizes are smaller than what can be found in the ALSPAC data, but the authors never discuss how larger sample sizes here (beyond 1,000-2,000 cases) would improve the LCA in question.
2. The authors discuss three dimensions of religiosity at the start of their article in the literature review (beliefs, feelings and behaviour), but only include a limited number of religiosity measures in their LCA, capturing only a few aspects of conventional religiosity: more so on belief in God, and a bit on forms of religious feelings and behaviour. Although the authors argue at the end of their article that their Moderately Religious category could be the equivalent of a spiritual but not religious group, no measures of less conventional spiritualities were included in the study’s LCA. Consequently, the study’s findings do not support this statement by the authors in their Discussion section. It is also not clear why the authors refer to their categorization as one of religious beliefs a number of times throughout their article, when they do have one measure of religious behaviour (attendance) included in the LCA as well.
3. The results of this study’s LCA seem to produce categories along a high/low religiosity continuum, which is reinforced by the authors’ interpretation of the four classes generated from the LCA. The scores of each of the five measured variables seem to decline relatively linearly from the Highly Religious to the Atheist generated categories. Consequently, there does not seem to be any substantive reason why a four-category religiosity measure here would be an improvement over a continuous religiosity scale generated from more simplistic statistical methods such as from a summative scale or from a Factor Analysis (as previous research has done). There are some more minor technical advantages to using LCA or Factor Analysis over a summative scale. Yet, from what is discussed of the study’s results, the substantive impact on the generated measure would only be very minor and probably not affect the results much. I would have liked to see a discussion by the authors of how their four categories may have deviated from a linear continuum of high to low religiosity (I do not see it in the results provided), so as to greater justify the use of the LCA in this instance.  

**Areas for future research:**

- Despite the limitations of this study, I do believe LCA is a statistical technique that can make many contributions to the social scientific study of religion, mainly by developing better measures of religiosity, spirituality and secularity that better reflect realities in the field. Researchers should continue to explore the use of this statistical technique.
- We need to include less conventional survey measures of religiosity, spirituality and secularity (not just belief in God, religious affiliation and frequency of religious service attendance) in LCAs to take full advantage of this statistical technique, and to better capture the multidimensional nature of these phenomena.
- We also need to begin running LCAs with cross-national samples, to see how consistent religiosity/spirituality/secularity categories are across cultural contexts.

**Further reading:**

- For another example of LCA to capture religiosity and spirituality categories, see Wilkins-Laflamme, Sarah. 2022. *Religion, Spirituality and Secularity among Millennials: The Generation Shaping American and Canadian Trends*. New York: Routledge.

Is the work clearly and accurately presented and does it cite the current literature?

Yes

If applicable, is the statistical analysis and its interpretation appropriate?

Yes

Are all the source data underlying the results available to ensure full reproducibility?

Yes

Is the study design appropriate and is the work technically sound?

Partly

Are the conclusions drawn adequately supported by the results?

Partly

Are sufficient details of methods and analysis provided to allow replication by others?

Yes

Reviewer Expertise:

Quantitative methods; Sociology of religion

I confirm that I have read this submission and believe that I have an appropriate level of expertise to state that I do not consider it to be of an acceptable scientific standard, for reasons outlined above.

## References

[1] : Religion, Spirituality and Secularity among Millennials.2022; 10.4324/9781003217695 10.4324/9781003217695

# Response

**Published:** 2025-04-08

**Authors:** Isaac Halstead

**Affiliations:** University of Bristol, UK

R2  This article uses Latent Class Analysis (LCA) with UK ALSPAC data from the early 1990s to generate a more comprehensive measure of religiosity than has been seen in some past research. This study makes a modest contribution to the social scientific study of religion. LCA is still rare in the sociology and psychology of religion disciplines, and so an example of the technique in the field is a relevant methodological research note. Some of the study’s findings are also interesting. 

 This said, there are a few key issues with this study that make it, in my opinion, not appropriate to be indexed as a full research article. These include the age of the data, the limited measures included in the LCA, and the lack of discussion of why the LCA in this case produces a better-quality measure than simpler techniques such as a summative scale or a Factor Analysis would have done. I detail these three main issues below. 

1. Although it is normal to have a time gap between survey data collection and when researchers are able to index findings from these survey data, 30 years is a bit much. The religious landscape has changed a lot in the UK since the early 1990s (when the ALSPAC data was collected). Relationships between religiosity and socioeconomic variables have potentially changed since then, especially with more religiously diverse immigration to the country since the 1990s and nonreligious populations expanding into more demographic groups as nonreligion has become more normalized in UK society and nonreligious populations have grown in size over the past 30 years. There is no reason provided by the authors why this early 1990s ALSPAC data is better than any more recent data coming from the UK for the purposes of their analyses in this study. More recent waves of the British Social Attitudes survey, the European Values Study and the International Social Survey Programme religion module for example all include more demographically representative samples, better geographic coverage of the UK, data collected in the 2010s and 2020s, and multiple measures of religiosity (more religiosity measures than the ALSPAC data in fact). Yes, their sample sizes are smaller than what can be found in the ALSPAC data, but the authors never discuss how larger sample sizes here (beyond 1,000-2,000 cases) would improve the LCA in question. 

Thank you for this feedback. This paper is intended to provide a set of latent classes at a time point that is causally before the mother gives birth, allowing better causal inferences to be drawn about subsequent child and parent outcomes, which may be influenced by parenthood by taking advantage of the prospective nature of this cohort study and examining mothers and offspring during their lifecourse. These classes would then be used to examine a range of psychological and epidemiological outcomes, while addressing confounding and causality. Regarding the suitability of the measures, other studies lack the broad range of mental and physical health, and behavioural measures available as outcomes, as well as the psychological, socioeconomic, and epidemiological confounders, all of which are available for both offspring and parents. Combined, this allows us to prospectively examine associations over the life course. While examining the effect of societal attitudes towards religion and its decline over time is an interesting topic, it is not the aim of the present study and is more sociological than psychological or epidemiological, which are the disciplines our approaches and analyses are informed by.  We have added the following to remove any ambiguity about the aim of the present study:  The intention of the present study is to provide a set of latent classes for use in examining prospective associations, in outcomes relating to both parent and offspring.   

1. The authors discuss three dimensions of religiosity at the start of their article in the literature review (beliefs, feelings and behaviour), but only include a limited number of religiosity measures in their LCA, capturing only a few aspects of conventional religiosity: more so on belief in God, and a bit on forms of religious feelings and behaviour. Although the authors argue at the end of their article that their Moderately Religious category could be the equivalent of a spiritual but not religious group, no measures of less conventional spiritualities were included in the study’s LCA. Consequently, the study’s findings do not support this statement by the authors in their Discussion section. It is also not clear why the authors refer to their categorization as one of religious beliefs a number of times throughout their article, when they do have one measure of religious behaviour (attendance) included in the LCA as well. 

  Thank you for this feedback. Religiosity was just one aspect of many that were covered in the ALSPAC questionnaires. ALSPAC was not designed specifically as a study of religiosity, but a range of factors affecting health and development. There were limited religiosity items available at this timepoint. However, this is still a greater range of items than often found in psychology of religion domains (e.g. in research examining the association between religiosity and mental health). The point about spirituality in the discussion is speculative, as spirituality is only available in the more recent measures from 2018 onwards. Hopefully the more recent data collections will be able to shed some light on the possibilities suggested in this study. 

   

1. The results of this study’s LCA seem to produce categories along a high/low religiosity continuum, which is reinforced by the authors’ interpretation of the four classes generated from the LCA. The scores of each of the five measured variables seem to decline relatively linearly from the Highly Religious to the Atheist generated categories. Consequently, there does not seem to be any substantive reason why a four-category religiosity measure here would be an improvement over a continuous religiosity scale generated from more simplistic statistical methods such as from a summative scale or from a Factor Analysis (as previous research has done). There are some more minor technical advantages to using LCA or Factor Analysis over a summative scale. Yet, from what is discussed of the study’s results, the substantive impact on the generated measure would only be very minor and probably not affect the results much. I would have liked to see a discussion by the authors of how their four categories may have deviated from a linear continuum of high to low religiosity (I do not see it in the results provided), so as to greater justify the use of the LCA in this instance.  

 We are glad you agree with our point regarding them cohering into a broad continuum of low to high religiosity. If we rely upon certainty in belief in a higher power, it might seem that it's a linear change. However, some of the differences between the moderately and highly religious classes are contingent on behavioural and social factors (e.g. receiving help from religious sources), which provides an insight into the way these classes qualitatively differ. Furthermore, certainty/uncertainty in the (non)existence of a higher power represents a qualitatively different way of perceiving reality. Perhaps it is a limitation of the subjective labels that we imposed on the classes, but we attempted to provide a broad description of each class, despite them having varied conditional probabilities.

# Reviewer report: Reviewer response for version 1

**DOI:** 10.21956/wellcomeopenres.19915.r57537

**Published:** 2023-06-22

**Authors:** Telli Davoodi

**Affiliations:** 1 Wheelock College of Education & Human Development, Boston University, Boston, USA; 2 Princeton University, Princeton, USA

The paper uses a survey of a few religious belief and behavior items among a cohort of UK mothers and their partners to derive different latent classes of religiosity. These classes are briefly described and explored in association with socioeconomic indicators.

 I believe that this paper addresses an interesting question and could potentially contribute to the literature by proposing new items of religious belief and behavior to include in batteries of religiosity in survey research. However, there are important limitations that I think need to be addressed within the scope of the paper. Below, I list my concerns: 

- The paper offers no justification for why the specific items from Table 2 were included in the study; A specific framework would be helpful to understand why these particular belief and behavior items were selected given that there is a very wide range of items capturing the many dimensions of religiosity available to choose from.
- In the "Statistical Analyses" section, when the authors say that they carried out a latent class analysis on selected "religious belief" items, do they mean that they excluded the behavior items from Table 2 (e.g., frequency of worship; asking religious leaders for help). 
- The way the classes are named does not present the whole story. For example, the "Moderately Religious" group seems to have strong religious "beliefs" but not strong patterns of religious participation or engagement. Or in the "Atheist" group, almost everyone reports "occasional" religious attendance, which is somewhat at odds with how most atheists behave, I believe. Maybe this latter point is a consequence of how the options for the religious attendance question were recoded; specifically, why is there no "no attendance" option? 
- Overall, I recommend a naming convention that tells us a bit more about how the different groups differ with respect to their religious beliefs and practices. 
- I'm not sure what the numbers on Table 4 represent. Are the regression coefficients? Or p-values? Please specify on the table. 
- Table 5 and the discussion of the results presented in the table are not clear. For example, in the logistic regressions that tested the association between socioeconomic indicators and class, were all socioeconomic indicators entered at once (financial hardship, major financial problems, home ownership, and car access)? Did these models control for age and manual/non-manual class? Was there one model per latent class? When describing comparisons (e.g., " *the highly religious classes possessed a higher proportion of....*" what are we comparing to what? I take it that for example, in the first sentence under " *Associations between sociodemographic indicators and the latent class...*" section, we are comparing different levels of education, etc. *within *the highly religious class, rather than *between *the highly religious class and the other classes. This isn't clear from the write-up, however.
- A related point to the above comment is that most financial challenges seem to be related to whether the participant is from a manual or a non-manual occupational category, rather than their latent religiosity class. Can the authors address this point with the models or do they need to run additional models?
- I believe the authors need to discuss, in addition to the limitations they already discuss, the fact that the mothers in particular may be biased in this specific time of their lives because they are going through a unique experience where many physical and emotional changes take place potentially contributing to both their beliefs and behaviors with regards to the supernatural/religious/spiritual. 

Is the work clearly and accurately presented and does it cite the current literature?

Partly

If applicable, is the statistical analysis and its interpretation appropriate?

Partly

Are all the source data underlying the results available to ensure full reproducibility?

Yes

Is the study design appropriate and is the work technically sound?

Yes

Are the conclusions drawn adequately supported by the results?

Partly

Are sufficient details of methods and analysis provided to allow replication by others?

No

Reviewer Expertise:

Social and religious cognition, taking cross-cultural and developmental approaches.

I confirm that I have read this submission and believe that I have an appropriate level of expertise to state that I do not consider it to be of an acceptable scientific standard, for reasons outlined above.

# Response

**Published:** 2025-04-08

**Authors:** Isaac Halstead

**Affiliations:** University of Bristol, UK

R1  The paper uses a survey of a few religious belief and behavior items among a cohort of UK mothers and their partners to derive different latent classes of religiosity. These classes are briefly described and explored in association with socioeconomic indicators. 

 I believe that this paper addresses an interesting question and could potentially contribute to the literature by proposing new items of religious belief and behavior to include in batteries of religiosity in survey research. However, there are important limitations that I think need to be addressed within the scope of the paper. Below, I list my concerns:  

- The paper offers no justification for why the specific items from Table 2 were included in the study; A specific framework would be helpful to understand why these particular belief and behavior items were selected given that there is a very wide range of items capturing the many dimensions of religiosity available to choose from. 

These items were included because at this time point (antenatal to 9 years approximately 1990 to 1999), there was a more limited range of available measures, compared to later data collections (27 years onward). Only later waves of data collection had a broader range of measures. 

   

- In the "Statistical Analyses" section, when the authors say that they carried out a latent class analysis on selected "religious belief" items, do they mean that they excluded the behavior items from Table 2 (e.g., frequency of worship; asking religious leaders for help).  

Thank you for pointing this out, we have now differentiated between religiosity and religious belief in several places in the manuscript as this was confusing.   

- The way the classes are named does not present the whole story. For example, the "Moderately Religious" group seems to have strong religious "beliefs" but not strong patterns of religious participation or engagement. Or in the "Atheist" group, almost everyone reports "occasional" religious attendance, which is somewhat at odds with how most atheists behave, I believe. Maybe this latter point is a consequence of how the options for the religious attendance question were recoded; specifically, why is there no "no attendance" option?  

These classes provide a ‘soft assignment’ where membership is modelled probabilistically, rather than forcing member to one class alone. The names of the classes are also entirely subjective, chosen by the researcher to best describe the pattern of responses.  In cases such as the Atheist group reporting occasional attendance, this is likely due to attendance at weddings, funerals, or other events that may not be an indicator of their own religious belief. We have now included a table that clearly links the label we assigned to the class to the conditional probabilities of the classes (see Table 3).  We have addressed this by adding the following:   Based on the conditional probabilities, we assigned subjective labels that we felt provided a broad summary of the class.  

   Overall, I recommend a naming convention that tells us a bit more about how the different groups differ with respect to their religious beliefs and practices.   We have attempted to address this in the change made above. 

 'm not sure what the numbers on Table 4 represent. Are the regression coefficients? Or p-values? Please specify on the table.  Thank you for pointing this out, we have now made it clear in the caption for the table that they are regression coefficients 

    Table 5 and the discussion of the results presented in the table are not clear. For example, in the logistic regressions that tested the association between socioeconomic indicators and class, were all socioeconomic indicators entered at once (financial hardship, major financial problems, home ownership, and car access)?   We have also made it clear we were looking at the unadjusted proportions in each class:  All these associations were unadjusted for confounders.  Did these models control for age and manual/non-manual class?   No  Was there one model per latent class?   There was one model for each outcome.  When describing comparisons (e.g., "the highly religious classes possessed a higher proportion of...." what are we comparing to what? I take it that for example, in the first sentence under "Associations between sociodemographic indicators and the latent class..." section, we are comparing different levels of education, etc. within the highly religious class, rather than between the highly religious class and the other classes. This isn't clear from the write-up, however. 

   

- Thank you for this feedback, we have amended the relevant sections to indicate what they are in comparison to. A related point to the above comment is that most financial challenges seem to be related to whether the participant is from a manual or a non-manual occupational category, rather than their latent religiosity class. Can the authors address this point with the models or do they need to run additional models? 

This is an interesting point. However, we were just wanting to provide some basic information regarding the makeup of each class, as the intention of the paper was descriptive. This topic could be addressed by its own, more extensive paper. 

- I believe the authors need to discuss, in addition to the limitations they already discuss, the fact that the mothers in particular may be biased in this specific time of their lives because they are going through a unique experience where many physical and emotional changes take place potentially contributing to both their beliefs and behaviors with regards to the supernatural/religious/spiritual.  

This is a good point, we have added the following text:  Furthermore, there is the possibility that being pregnant may cause changes in a mother’s religiosity or spirituality, which may bias their responses at the time period examined in this study.
